# Data for professional socialization and professional commitment of nursing students — A case study: Kermanshah University of Medical Sciences, Iran

**DOI:** 10.1016/j.dib.2018.11.088

**Published:** 2018-11-22

**Authors:** Yahya Safari, Nasrin Yoosefpour

**Affiliations:** Research Center for Environmental Determinants of Health (RCEDH), Kermanshah University of Medical Sciences, Kermanshah, Iran

**Keywords:** Socialization, Professional commitment, Students, KUMS

## Abstract

Nursing students by professional socialization could catch the necessary professional commitment in this carrier. In addition, by solving the problem and having a specific and certain discipline could have act as a nurse. The aim of this study is evaluating the relation between professional socialization and professional commitment of nursing students in Kermanshah University of medical sciences (KUMS), Kermanshah, Iran. This research is a descriptive-analytical study that the investigated population were all the nursing students of KUMS, which were 80 persons and all of them were considered as a samples of this study. The collecting data were the professional socialization tool of Chao et al. (1994) and the professional commitment scale of Mayer and Alen (1996). The raw data analyzed by SPSS software (Ver.21). The Spearman test results have showed that there was no significant relation between professional socialization and professional commitment in nurses. The results of Spearman test for evaluating the relation of demographic variable and professional commitment was showed that there was no significant relation between gender, marital status and individuals age with their professional socialization. The overall results of this research was represent the weak relation between professional socialization and professional commitment of nursing students in KUMS, which this fact could decrease the caring quality efficiency. Therefore, the authorities must take necessary measures to accelerate the process of socialization and professional status of nurses.

**Specifications table**

**Table:** 

Subject area	Social sciences
More specific subject area	Health sciences
Type of data	Tables
How data was acquired	In this descriptive and analytical study, the correlation between professional socialization and professional commitment of nursing students of KUMS has been investigated. The statistical population of this study consisted of all Nursing students of KUMS (80 persons) that all of them were considered as a samples of this study. The data collection method were by standard questionnaires that included the professional socialization tool of Chao et al. (1994) and the professional commitment scale of Mayer and Alen (1996). The obtained raw data analyzed by SPSS software (Ver.21).
Data format	Raw, analyzed
Experimental factors	Unemployment during the education and non-transference or not being a guest at the KUMS, which the study was done in it, is the criteria for entering in present research.
Experimental features	The relationship between professional socialization and professional commitment of nursing students was evaluated using linear regression test.
Data source location	Kermanshah, Iran
Data accessibility	Data are included in this article
Related research article	M.Shali, S.Joolaee, A.Hooshmand, H.Haghani, H. Masoumi, The Relationship between Incidence of Patient Falls and Nurses’ Professional Commitment, Hayat. 22(2016)27–37 [1].

**Value of the data**

• The process of professional socialization in nursing students can help plan for the ability of nursing students to be successful in the clinical setting in order to improve the health system and perform their duties better. The data of present study evaluate the correlation between professional socialization and professional commitment of nursing students of KUMS.
• So far, such a study has not been done in KUMS, Therefore, the results of this study can be useful for students of this university.
• The data of this study could be the basis for doing similar studies in other medical sciences universities in Iran.
• The analyzed data of this study was showed that correlation between professional socialization and professional commitment of nursing students of KUMS is poor, therefore, the authorities must take necessary measures to accelerate the process of socialization and professional status of nurses.

## 1 Data

In this study 80% person have participated which from this number 70 percent were girl students and 12% were married. The average age of the students’ were 25.01 with the standard deviation of 3.55 year with the age range of 19–38 year (Table 1).

**Table 1:** Frequency distribution of demographic variables in research units.

**Variables**		**Frequency**	
		**Number**	**Percent**
Gender	Male	24	30
	Female	56	70
Marital status	Single	68	85
	Married	12	15

The descriptive information of professional socialization and professional commitment have present in Table 2, Table 3 with its subscales. The average and standard deviation of the professional socialization score is 46.76 ± 8.96 with the range of the 49–127 and the professional commitment average score is 94.53 with the standard deviation of 16.71. The change range score of professional commitment were 49–127.

**Table 2:** The mean and standard deviation of professional commitment and professional socialization with its subscales.

**Variables**	**Reliability coefficient (Cronbach׳s alpha)**	**Overall**			**Mean score for each items**
		**Mean±S.D**	**Min**	**Max**	
**Professional commitment**	0.76	94.53±16.71	49	127	3.94
• Emotional commitment	0.81	29.21 ±6.76	12	42	3.65
• Continuous commitment	0.73	31.60±6.84	8	48	3.95
• Normative commitment	0.74	33.72±8.83	14	52	4.21
**Professional socialization**	0.84	66.67±8.92	45	83	3.33
• Education	0.86	16.66±3.30	5	25	3.33
• Understanding	0.83	14.67±3.02	7	22	2.93
• Staff support	0.85	18.01±4.48	8	25	3.60
• Future prospects	0.82	15.32±3.14	5	25	3.06

**Table 3:** The comparison professional commitment and professional socialization with its subscales between men and women.

**Variables**	**Men**			**Women**			**p**
	**Mean±S.D**	**Min**	**Max**	**Mean±S.D**	**Min**	**Max**	
**Professional commitment**	96.01±15.92	59	127	91.08±18.32	49	124	0.229
• Emotional commitment	29.85±6.97	15	42	27.70±6.13	12	36	0.195
• Continuous commitment	31.19±7.26	8	48	32.54±5.77	22	43	0.424
• Normative commitment	34.96±8.55	18	52	30.83±8.98	14	50	0.055
**Professional socialization**	64.23±9.46	45	82	65.70±7.59	50	83	0.464
• Education	16.76±3.63	5	25	16.41±2.41	11	20	0.666
• Understanding	14.62±3.19	7	20	14.79±2.63	11	22	0.823
• Staff support	18.17±4.96	8	25	17.62±3.17	12	25	0.552
• Future prospects	14.66±2.92	5	21	16.87±3.15	12	25	0.003

The results of Spearman test have showed that there was no significant statistical relation between professional socialization and professional commitment in nurses. In addition the analysis of this test results have showed that there was no significant relation between the professional socialization component and professional commitment of nurses.

According to Table 4 the results of Spearman test evaluation for assessing the correlation between nurses׳ professional socialization with professional commitment and its components have showed that there was no significant between these variables.

**Table 4:** The correlation between nurses׳ professional socialization with professional commitment and its components.

**Variables**	**Professional commitment**		**Emotional commitment**		**Continuous commitment**		**Normative commitment**	
	**r**	**P**	**r**	**P**	**r**	**P**	**r**	**P**
**Professional socialization**	0.099	0.381	−0.016	0.888	0.087	0.441	0.088	0.436
• Education	0.054	0.636	0.033	0.769	0.092	0.418	0.066	0.561
• Understanding	0.035	0.756	0.010	0.930	0.087	0.441	0.052	0.646
• Staff support	0.180	0.110	0.054	0.637	0.103	0.363	0.161	0.153
• Future prospects	−0.063	0.577	−0.129	0.255	−0.133	0.240	−0.019	0.867

According to the Table 5 evaluation of nurse׳s demographic variables and socialization have showed that there was no significant relation between individual׳s gender, marital status, age and professional socialization.

**Table 5:** Correlation between the score of professional socialization and professional commitment in terms of demographic variables.

**Variables**	**Professional commitment**		**Professional socialization**	
	**r**	**P**	**r**	**P**
Gender	+0.102	0.367	−0.073	0.522
Marital status	+0.060	0.598	0.130	0.294
Age	+0.025	0.827	0.036	0.754

## 2 Study design, materials and methods

This is a descriptive-analytical study. The statistical population include all the university students of apprentices’ level in Kermanshah medical science university, which were 80 persons. The sampling have done by census method [2], [3], [4], [5], [6], [7], [8], [9], [10]. For analyzing the data the descriptive statistics (average, standard deviation) and Pearson correlation coefficient test have used. Unemployment during the education and non-transference or not being a guest at the KUMS, which the study was done in it, is the criteria for entering in present research. In the present study for collecting data, the standard questionnaires were used.

Although both questionnaires are standard and are valid in the previous studies in terms of validity and reliability. However, the reliability of the questionnaires in the current statistical society is also estimated and the Cronbach׳s alpha coefficient for the professional socialization questionnaire was 0.91 and for social commitment questionnaire 0.86 was obtained. The reliability coefficient (Cronbach׳s alpha) for all dimensions related to professional commitment and professional socialization reported in Table 2.

A. Chao et al. (1994) 48 questions questionnaires: this questionnaire have evaluated four field of measurement and recognition of professions, professional skill, organizational politics and management and interpersonal communication. In this research the students should declared his agreement rate about each choices with selecting a number between one (showed very low respond) to seven (showed very high respond). The obtained score of each question have summed and the total score of the questionnaire is varied from 48–336. According to the questionnaire instruction the score of 48–107 have considered very low, the score of 108–162 low, 163–221 average, 22–278 high and 279–336 very high in terms of professional socialization [11].
B. The professional commitment scale: the professional commitment scale have consist of 24 question which have made and validated by Mayer and Allen (1996), question 1–8 are related to emotional commitment subscale and question 9–16 are for continual commitment scale, questions 17–24 are related to the norm commitment scale and include 8 question. Scoring of the professional commitment scale is from 1–7, mean totally agree (7) totally disagree (1). In this scale, the score range of scale is 24–168. It means the least score is 24 and the highest score is 168 [1,12 and 13].

After the implementation of the questionnaire on the statistical samples and collecting data for analysis, the indexes and descriptive statistical method have used which include: frequency, percentage, average, standard deviation, inferential statistic such as Spearman correlation coefficient. The distribution normality of obtained raw data was analyzed by one-Sample Kolmogorov-Smirnov Test by SPSS.21 software (SPSS Inc. Chicago, IL, USA) that result of that presented in Table 6. The data distribution for the some variables including “education”, “understanding”, “future prospects” and “emotional commitment” was non-normal but for other investigated variables, was normal (Table 6).

**Table 6:** The investigation of distribution normality of obtained raw data by one-Sample Kolmogorov-Smirnov.

**Variables**		**Education**	**Understanding**	**Staff support**	**Future prospects**	**Professional socialization**	**Emotional commitment**	**Continuous commitment**	**Normative commitment**	**Professional commitment**
**N**		80	80	80	80	80	80	80	80	80
**Normal Parameters**^a^^**,**^^b^	**Mean**	16.67	14.67	18.02	15.33	64.68	29.22	31.61	33.72	94.53
	**S.D**	3.30	3.02	4.49	3.14	8.92	6.77	6.84	8.83	16.71
**Most Extreme Differences**	**Absolute**	.120	.118	.090	.138	.053	.110	.063	.080	.068
	**Positive**	.093	.056	.089	.138	.053	.069	.050	.080	.045
	**Negative**	−.120	−.118	−.090	−.108	−.050	−.110	−.063	−.054	−.068
**Test Statistic**		.120	.118	.090	.138	.053	.110	.063	.080	.068
**Skewness**		1.676	1.525	1.452	1.661	1.872	1.523	1.655	1.554	1.690
**Kurtosis**		1.717	1.212	1.328	1.824	1.525	1.487	1.773	1.742	1.563
**P value (2-tailed)**		.006^c^	.008^c^	.164^c^	.001^c^	.200^c^^,^^d^	.018^c^	.200^c^^,^^d^	.200^c^^,^^d^	.200^c^^,^^d^

a Test distribution is Normal.

b Calculated from data.

c Lilliefors Significance Correction.

d This is a lower bound of the true significance.
