# Supplementary material for: Data for professional socialization and professional commitment of nursing students — A case study: Kermanshah University of Medical Sciences, Iran
Source: Data Brief. 2018 Nov 22;21:2224–9. doi: 10.1016/j.dib.2018.11.088 (PMC6276542; doi:10.1016/j.dib.2018.11.088)
Supplement: Supplementary file 1 — Supplementary material [file mmc1.doc]

Dear editor(s) of Data in Brief journal

Greetings!

We would be grateful if you would consider the attached manuscript “**Data for Correlation between Professional Socialization and Professional Commitment of Nursing Students- A Case Study: Kermanshah University of Medical Sciences, Iran”** for publication in valuable Journal Data in Brief. We declare that this manuscript, or part of it, has neither been published nor is currently under consideration for publication by any other journal. We also declare that we have read the manuscript and approved submission to Journal of Data in Brief. The Project was supported by Research Council of Kermanshah University of Medical Sciences for the financial support of this research (Grant Number: 96389), Iran. None of the authors of this paper have any conflicts of interest to declare.

Kind Regards,

Mrs. Nasrin Yoosefpour

***Corresponding author:**

E-mail address: Yoosefpour2018@gmail.com (N. Yoosefpour).

Tel: +0098-09181324798
